# General Therapeutics and Materia Medica

**Published:** 1858-01

**Authors:** 


ART. VII.—*General Therapeutics and Materia Medica; adapted for a Medical Text-Book; with Indexes of Remedies, and of Diseases and their Remedies.* By ROBLEY DUNGLISON, M.D., LL.D., Professor of Institutes of Medicine, etc., in Jefferson Medical College; formerly Professor of Materia Medica and Therapeutics in the Universities of Virginia and Maryland, and in Jefferson Medical College. Two vols. 8vo, pp. 542 and 539. Sixth edition, revised and improved, with one hundred and ninety-three illustrations. Blanchard & Lea, Philadelphia, 1857.

IN announcing a new edition of Dr. Dunglison's General Therapeutics and Materia Medica, we have no new words of commendation to bestow upon a work whose merits have been heretofore so often and so justly extolled. It must not be supposed, however, that the present is a mere reprint of the previous edition; the character of the author for laborious research, judicious analysis, and clearness of expression, is fully sustained by the numerous additions he has made to the work, and the careful revision to which he has subjected the whole.

